# Critical control point-based assessment and intervention of ochratoxin A risk in Angelicae Gigantis Radix production

**DOI:** 10.3389/fmicb.2022.952628

**Published:** 2022-10-21

**Authors:** Juil Kim, Tae Jin An, Yuseok Moon

**Affiliations:** ^1^Laboratory of Mucosal Exposome and Biomodulation, Department of Integrative Biomedical Sciences and Biomedical Research Institute, Pusan National University, Yangsan, South Korea; ^2^Department of Herbal Crop Research, National Institute of Horticultural and Herbal Science (NIHHS), Eumseong, South Korea; ^3^Graduate Program of Genomic Data Sciences, Pusan National University, Yangsan, South Korea

**Keywords:** mycotoxins, postharvest, Angelicae Gigantis Radix, thermal drying, critical control points

## Abstract

Improperly practiced postharvest procedures can pose mycotoxin-related risks during medicinal herb production. As a health food material with pharmacological activities, Angelicae Gigantis Radix (AGR) has been extensively used in oriental medicine or functional foods. Compared with the official protocol, conventional practices were investigated for provisional critical control points (CCPs) in terms of ochratoxin A (OTA) contamination. Conventional practices include field-drying, which was associated with increased fungal exposure. Compared with conventional methods, the washing process in the official protocol was not advantageous for reducing OTA contamination in final products. Instead, drying was examined to assess the fungal growth risk during AGR production. To reduce the energy cost, product overload and shortened drying time could lead to failure in controlling fungal overgrowth and subsequent OTA production. In particular, inner parts of the load contained a higher OTA content than outer parts close to the heat outlet of the dryer. Improper thermal drying of loads allowed the growth of ochratoxigenic species during AGR production. Collectively, non-field-drying and optimally loaded thermal drying are easy preventive actions in key CCPs that need to be well maintained to attenuate any further microbial risk. These assessments provide insights into good practice-based mycotoxin risk management in producing herbal medicinal crops and new cost-efficient appropriate interventions for small-scale farms.

## Introduction

Among broadly used herbal medicines, Angelicae Gigantis Radix (AGR; roots of *Angelica gigas*), also called Korean Angelica, has been extensively employed to treat patients in different clinical settings in Asia (Eum et al., 2005; Sohn et al., 2012). In particular, traditional medication using AGR has been extensively used as a supplementary herbal medicine against cardiovascular disorders, menstrual pain, anemia, and allergic diseases (Eum et al., 2005; Sohn et al., 2012; Park et al., 2015). Moreover, the anti-inflammatory activity of AGR, mediated by blocking different pro-inflammatory mediators, including cyclooxygenase, tumor necrosis factor-alpha, and nitric oxide, has been demonstrated in different inflammatory animal models (Shin et al., 2009, 2010). Network pharmacology-based mechanistic prediction suggests that AGR-mediated actions are associated with hematopoiesis-promoting activity against hematologic disorders (Lee et al., 2020).

The hazard analysis critical control point (HACCP) is a systemic approach that identifies, evaluates, and controls hazards at critical control points (CCPs), which are essential steps to prevent and eliminate food hazards and achieve acceptable safety levels for potential hazards. The HACCP of agricultural commodities builds on the foundations of well-established quality and safety management systems, such as good agricultural practices (GAPs) and good storage practices. Relatively time- and labor-consuming postharvest processes, including storage, drying, and farmer manufacturing, can increase the risk of postharvest mycotoxins, particularly considering production on small farms where guidelines for standard preparation are technically not well performed. Examples include a study on improved integrated mycotoxin management in adlay (Choi et al., 2014) or coffee production systems (Urbano et al., 2001; Lopez-Garcia et al., 2008), reporting various risk factors due to a lack of safety awareness, storage, and processing infrastructure to reduce fungal infection, and a long commercial chain from untechnified small-family farms.

Herbal medicines can be contaminated with a broad variety of toxic fungal metabolites, including mycotoxins from *Aspergillus* spp., *Penicillium* spp., and *Fusarium* spp. (Halt, 1998; Trucksess and Scott, 2008; Kosalec et al., 2009). However, naturally occurring mycotoxins limit the utilization of medicinal materials and present potential risks to human health. Numerous studies have reported mutagenic mycotoxins, such as aflatoxins and ochratoxin, in botanicals, such as ginseng, ginger, licorice, turmeric, and kava-kava in the United States, China, Argentina, India, and other countries (Tassaneeyakul et al., 2004; Ip and Che, 2006; Ardic et al., 2008; Katerere et al., 2008; Chen et al., 2010; Han et al., 2011). Although the European Union (EU) has established legal limits for major mycotoxins in various foodstuffs, official legislation is only available for aflatoxin and ochratoxin A (OTA) contamination in several medicinal herbs and spices, including *Curcuma longa* (turmeric), *Mystica fragrans* (nutmeg), *Pipper* spp. (pepper), and *Zingiber officinale* (ginger), limited to 15 μg/kg (Schrenk et al., 2020). Additional EU legislation limits for OTA have also been established for *Glycyrrhiza* spp. (licorice) and licorice extracts at 20 and 80 μg/kg, respectively. Despite their extensive use as complementary herbal medicines or functional food supplements, regulatory limits for AGR products have not been established globally. Before the regulatory setting, it is warranted to assess the agronomic practices including pre- and post-harvest processes in terms of microbial risk. There have been microbial concerns in AGR production. For example, some farms run the conventional field drying or many do not include a washing process owing to concerns regarding the loss of pharmacological components. In the present study, we aimed to address mycotoxin contamination during AGR production. In particular, we identified provisional CCPs during postharvest practices in small-scale farms where the OTA contamination risk was evaluated. Procedure-based assessments provide practical insights into mycotoxin risk management and appropriate reduction technologies in other crop production.

## Materials and methods

### Sampling

After 1^st^ year of sowing in mid-late April, the seedlings are cultivated but gradually enter a dormant period during the winter. In the spring, the seedlings that start to sprout again are prepared for transplantation. After the transplantation in the second-year spring, Angelica shoots grow again. When the temperature gradually decreases from September, the growth of the roots accelerates and gradually becomes fleshy. During this time, AGR can be harvested and processed into herbal medicines. In the present study, we evaluated the seedling transplanted to different types of fields based on the duration of continuous cropping in each region. All AGR samples were collected for fungal contamination on the first fall after transplantation. Preharvest fresh AGR samples (*n* = 31) were collected from different locations (Gangwon province, Korea) to measure mycotoxin contamination levels. In addition to the preharvest produce, postharvest samples were collected based on the postharvest process conditions such as the dying conditions (heated air drying or field-drying) and storage temperatures (25 or 4°C). Specific sample numbers in each post-harvest step were denoted in each figure legend. Each sample weighed 500–1,000 g and was ground to pass through a 0.5-mm sieve. All samples were stored at −20°C until mycotoxin analysis and parts of unfrozen samples were directly evaluated for mold analysis. Each sample was manually mixed in a sterile Ziplock^®^ plastic packet before analysis to ensure homogeneity. Moreover, the moisture content was determined by comparing the sample weights before and after drying out in a hot air oven at 105°C until a constant weight was attained.

### High-pressure liquid chromatography analysis

HPLC was performed to confirm the ELISA results of OTA in AGR. OTA extraction was performed by shaking AGR (20 g) with 100 ml of methanol–water (70,30, v/v) for 30 min. The obtained extract was filtered through Whatman filter paper (no. 1), and the filtrate was collected. The filtrate (7 ml) was then diluted with 28 ml dilution buffer (500 mm NaCl, 20 mm Tris pH 8.0, 20 mm EDTA, 1% TritonX-100, 0.1% sodium dodecyl citrate [SDS]). The diluted filtrate was introduced into an immunoaffinity column (IAC; Vicam, Watertown, MA, United States) for 1 h, and the column was washed with 20 ml of 0.1× phosphate-buffered saline. OTA was eluted from the IAC using 1 ml of methanol, and the eluate was collected and evaporated to dryness under nitrogen. Before HPLC analysis, the evaporated samples were dissolved in 200 μl of the mobile phase. OTA was identified by constant retention time and quantified by comparing peak areas of the standards in the mobile phase. The HPLC apparatus (Shimadzu, Kyoto, Japan) consisted of a quaternary pump, and a fluorescence detector was used with a stainless steel reverse-phase 300 × 3.9 mm C18 Supelco HPLC column (Supelco, Bellefonte, PA, United States). For OTA detection, the mobile phase consisted of acetonitrile, water, and acetic acid (50,50:1, v/v/v), passaged at a flow rate of 1 ml/min. OTA standard was obtained from Supelco (Bellefonte) and its standard working solution (100 *μ*g/kg) was prepared by nitrogen-based evaporation. The detector wavelengths were set at an excitation wavelength of 333 nm and an emission wavelength of 460 nm. Samples were confirmed using an LC/mass spectrometry-selected ion monitoring system (QSTAR XL Pro System; Life Technologies Korea, Seoul, Korea). A calibration curve was obtained using the linear least squares regression procedure of the peak area versus the concentrations (0.5, 1.0, 2,5, 5.0, 10.0, 25.0, 50.0, and 100 μg/kg OTA; Supplementary Figure 1A). The limit of detection (LOD) was 0.122 μg/kg, while the limit of quantitation (LOQ) was 0.485 μg/kg using the signal-to-noise approach, defined as per the concentration, resulting in a signal-to-noise ratio of approximately 3:  1 and 10:  1 for LOD and LOQ, respectively. Since there is no certified reference material available for AGR, 25 g OTA-free AGR samples were spiked with different concentrations of OTA (1, 5, 25 μg/kg) in three replicates. The method accuracy was calculated as the recovery (peak area of the spiked sample with standard solution divided by the peak area of the standard solution at the same concentration level) × 100%. The mean recoveries were 89.1–94.4% for OTA.

### Quantitation of fungi in AGR samples

Briefly, 1 g of finely ground AGR was suspended in 50 ml buffered saline peptone solution (pH 7.0, containing 0.1% Tween 80). Then, 100 μl of the sample solution was serially diluted to 10^−4^ and spread on potato dextrose agar (containing 0.5 mg/ml chloramphenicol) at 20°C for 5 days. For morphological observation of colony diameters, appearances, and pigmentations (Pitt, 2000), samples were grown on Czapek yeast autolysate agar (KisanBio, Seoul, Korea), malt extract agar (KisanBio), and yeast extract sucrose agar (KisanBio) *via* incubation at 25°C for 7 days.

### Identification of OTA-producing fungi in AGR samples

Isolated fungal DNA was amplified using primers for the internal transcribed spacer (ITS) region for all unknown molds (5’-TCC GTA GGT GAA CCT GCG G-3′ and 5’-TCC TCC GCT TAT TGA TAT GC-3′; Sang et al., 2013), β-tubulin (BT2) for *Aspergillus* and *Penicillium* species (5′-GGT AAC CAA ATC GGT GCT GCT TTC-3′ and 5’-ACC CTC AGT GTA GTG ACC CTT GGC-3′; Glass and Donaldson, 1995) and calmodulin (*CF*) for *Penicillium* species (5′-AGG CGG AYT CTY TGA CYG A-3′ and 5′-TTT YTG CAT CAT RAG YTG GAC-3′; Peterson and Multilocus, 2004; Peterson and Horn, 2009). The cDNA was amplified using Takara HS ExTaq DNA polymerase (Takara Bio, Shiga, Japan) in a MyCycler Thermal Cycler (Bio-Rad, Hercules, CA, United States) using the following parameters: denaturation at 94°C for 2 min followed by 35 cycles of denaturation at 98°C for 10 s, annealing at 52°C (ITS1), 58°C (BT2), or 54°C (*CF*) for 30 s, and elongation at 72°C for 45 s. The PCR products (600 bp ITS1 amplicon, 500 bp BT2 amplicon, and 650 bp *CF* amplicon) were purified using the Wizard PCT Prep Kit (Promega, Madison, WI, United States). Purified double-stranded PCR fragments were directly sequenced using the BigDye Terminator Cycle Sequencing Kit (Applied Biosystems, Foster City, CA, United States). The same primer sets used for PCR amplification were employed to sequence both DNA strands. Gel electrophoresis and data collection were performed on an ABI Prism 310 Genetic Analyzer (Applied Biosystems, Foster City, CA, United States).

### Setup of provisional CCPs during AGR preparation

**Figure 1:**
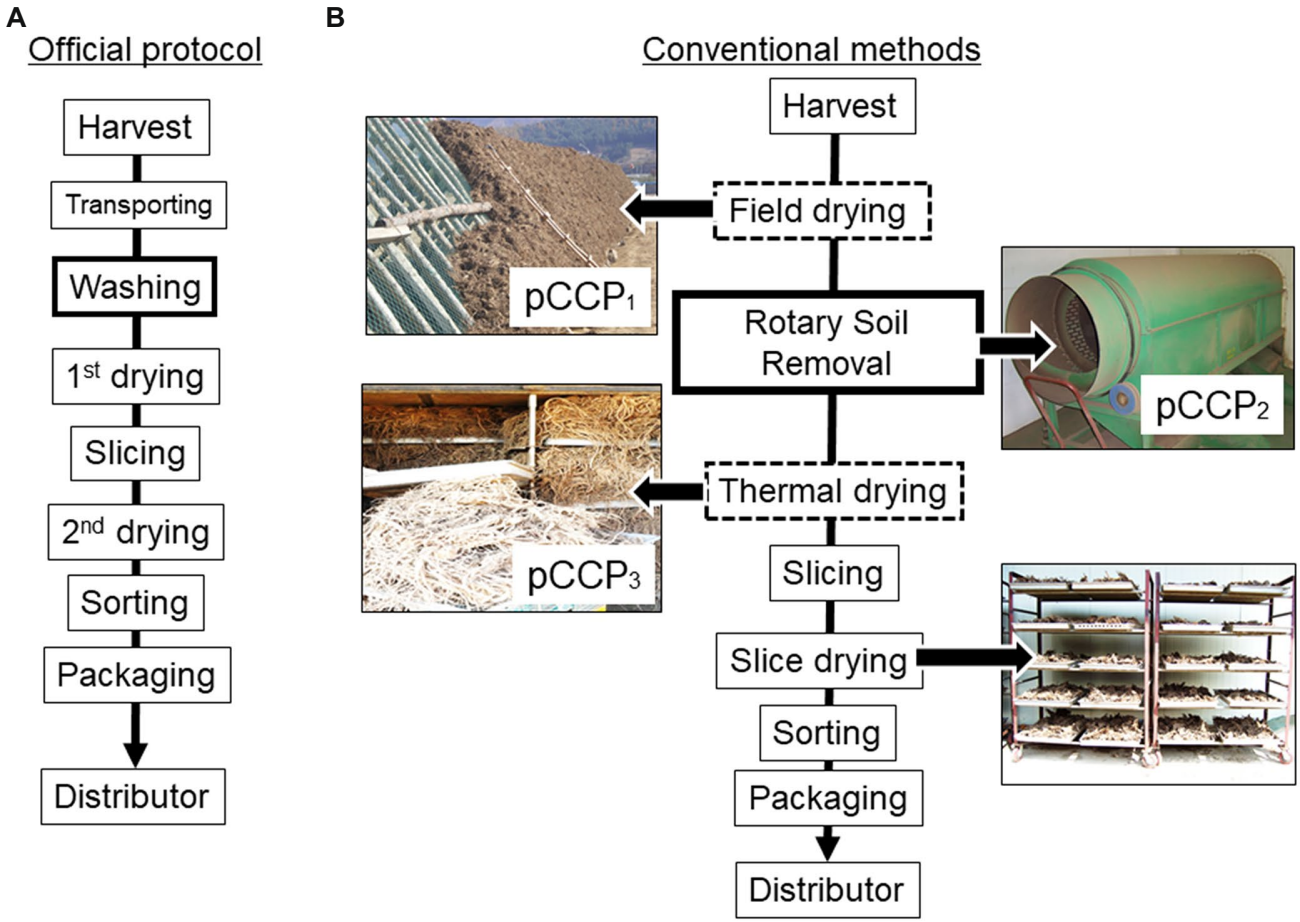
Comparison between the official protocol **(A)** and the conventional practice in AGR **(B)** with provisional critical control points (PCCPs) in AGR production. AGR, Angelicae Gigantis Radix.

Given that AGR production is primarily dependent on small-family farm-based practices, the official protocol of the postharvest process for AGR has been guided to maintain regular quality (Park et al., 2008). This includes the first drying immediately after washing, sorting, slicing, the second drying, and packaging as GAPs (Figure 1A). In particular, postharvest washing and double drying have been recommended as key processes for ensuring microbial safety. However, most farms carry out conventional practices, with several modifications during the postharvest processes of AGR production (Figure 1B). Briefly, the harvested produce is dried in open fields or directly dried in a thermal dryer without undergoing a washing process. Some portions of family farms still perform field-drying for several months to reduce the total moisture content in AGR, additionally performing thermal drying if only field-drying is insufficient. Recently, most farms have performed thermal drying immediately after harvesting. Given that field-dried AGR is directly exposed to the outdoor environment and is subsequently susceptible to microbial inoculation, the drying step was set as a provisional CCP (pCCP_1_). Moreover, the conventional procedure at most small-scale farms does not include a washing step owing to concerns regarding the loss of pharmacological components during the washing process, which has also been scientifically verified (Seong et al., 2018). Instead, the farms minimize the washing process or most attached soils are eliminated using a rotary soil remover; however, the risk of soil-borne microbial contamination and subsequent toxin production on the AGR surface may persist. Therefore, the soil removal step was set as the provisional CCP_2_. After drying, the steamed AGR was sliced and dried again in a thermal drier at 38–40°C. However, if an AGR shipment is delayed for a prolonged period by more than several months due to market price fluctuations, extended storage poses the risk of fungal growth and mycotoxin production. After the slicing step, the produce is transported and stored in agricultural product processing centers (APCs), which are nationally authorized facilities for local farmers. The storage facility of APCs controlled the temperature and moisture during storage to ensure the safety and quality of AGR transported from small-scale farms.

### Statistical analyses

Statistical analysis was performed as described in our previous report (Park et al., 2020; Kim and Moon, 2021). All statistical analyses were performed using GraphPad Prism v. 5.01 (GraphPad Software, La Jolla, CA, United States). For comparative analysis of the two data groups, the Student’s *t*-test was performed. For comparative analysis of multiple groups, data were subjected to analysis of variance (ANOVA), with the Newman–Keuls method employed as a *post-hoc* ANOVA.

## Results

### OTA contaminates AGR products during postharvest practices

**Figure 2:**
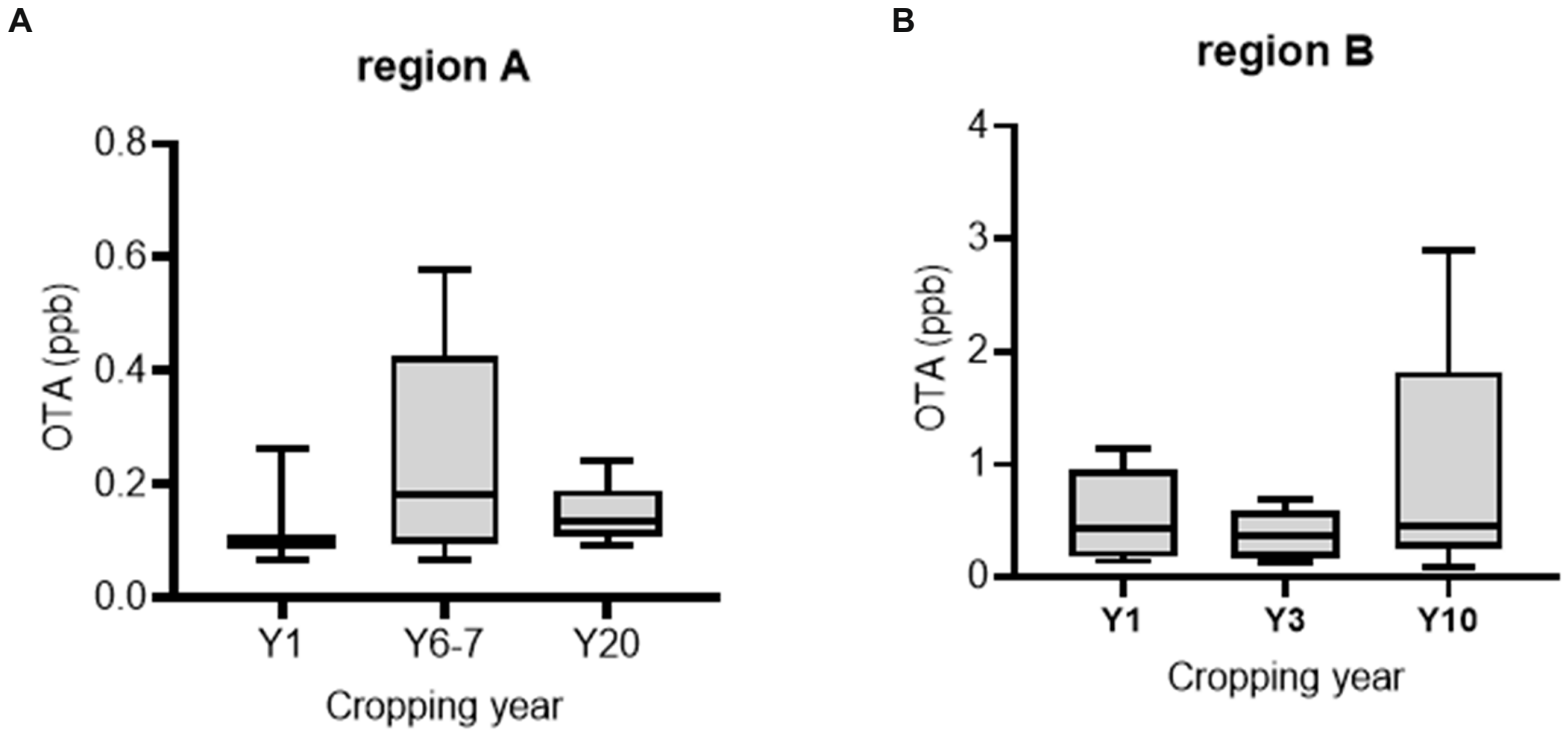
OTA levels in freshly harvested AGR from regions **A** (*n* = 5, 6, and 5 per group in order) and **B** (*n* = 5 per group). Results are shown as a box-and-plot with Tukey whiskers indicating the difference between the 75^th^ and 25^th^ percentiles with one-way ANOVA and the Newman–Keuls *post hoc* test (*p* = 0.05).

**Figure 3:**
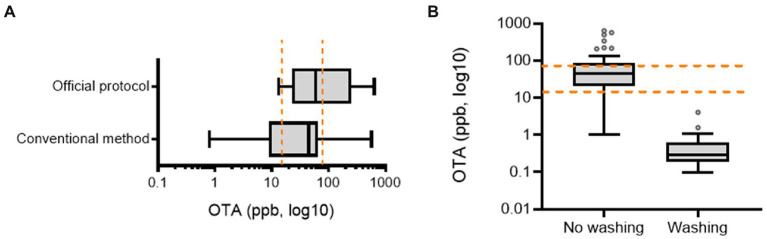
OTA levels in AGR products. **(A)** Comparison between official protocol- and conventional practice-based AGR products (*n* = 10 and 20 per group in order). **(B)** Effects of the washing process on OTA levels in AGR products (*n* = 20 per group). Results are shown as a box-and-plot with Tukey whiskers indicating the difference between the 75^th^ and 25^th^ percentiles with Student’s *t*-test (*p* = 0.05). AGR, Angelicae Gigantis Radix; OTA, ochratoxin A. The orange-colored dashed lines indicate the EU regulatory limits for OTA in medical crops (15–80 ppb).

Official postharvest protocol-guided AGRs were compared with those from conventional practices. First, we assessed the preharvest AGR produce as a potential source of OTA contamination. We collected fresh produce samples from two main AGR production regions in Korea (the highland regions 1 and 2 in Gangwon Province). Although most samples contained detectable OTA levels, these levels were lower than the EU regulatory limit for OTA in medical crops (15–80 ppb; Figures 2A,B). Moreover, AGRs from continuous cropping tended to contain higher OTA levels than those from the first-year cropping, although with marginal statistical significance. Next, we measured OTA levels during the postharvest procedure. As the official protocol includes a washing process postharvest, it may reduce the risk of microbial colonization or toxin production. In contrast to our prediction, half of the official protocol-based products were contaminated with high levels of OTA (˃80 ppb; Figure 3A). Although no significant difference in OTA levels was detected between the two practice-based products, most conventional practice-based products were within the level corresponding to the upper regulatory limit of the EU regulatory guide (80 ppm). Moreover, the conventional practice-based products were assessed for contamination with OTA immediately after the washing process which was originally excluded in conventional practice. However, the washing process of the products was effective in reducing OTA contamination (Figure 3B). Although the official practice run the washing process directly after harvest, the official guide-based products were contaminated with high OTA levels (Figure 3A). Therefore, it was necessary to investigate post-washing processes including the thermal drying that may increase the mycotoxin risk during the official as well as the conventional practices.

### OTA contaminates AGR products during the thermal process

**Figure 4:**
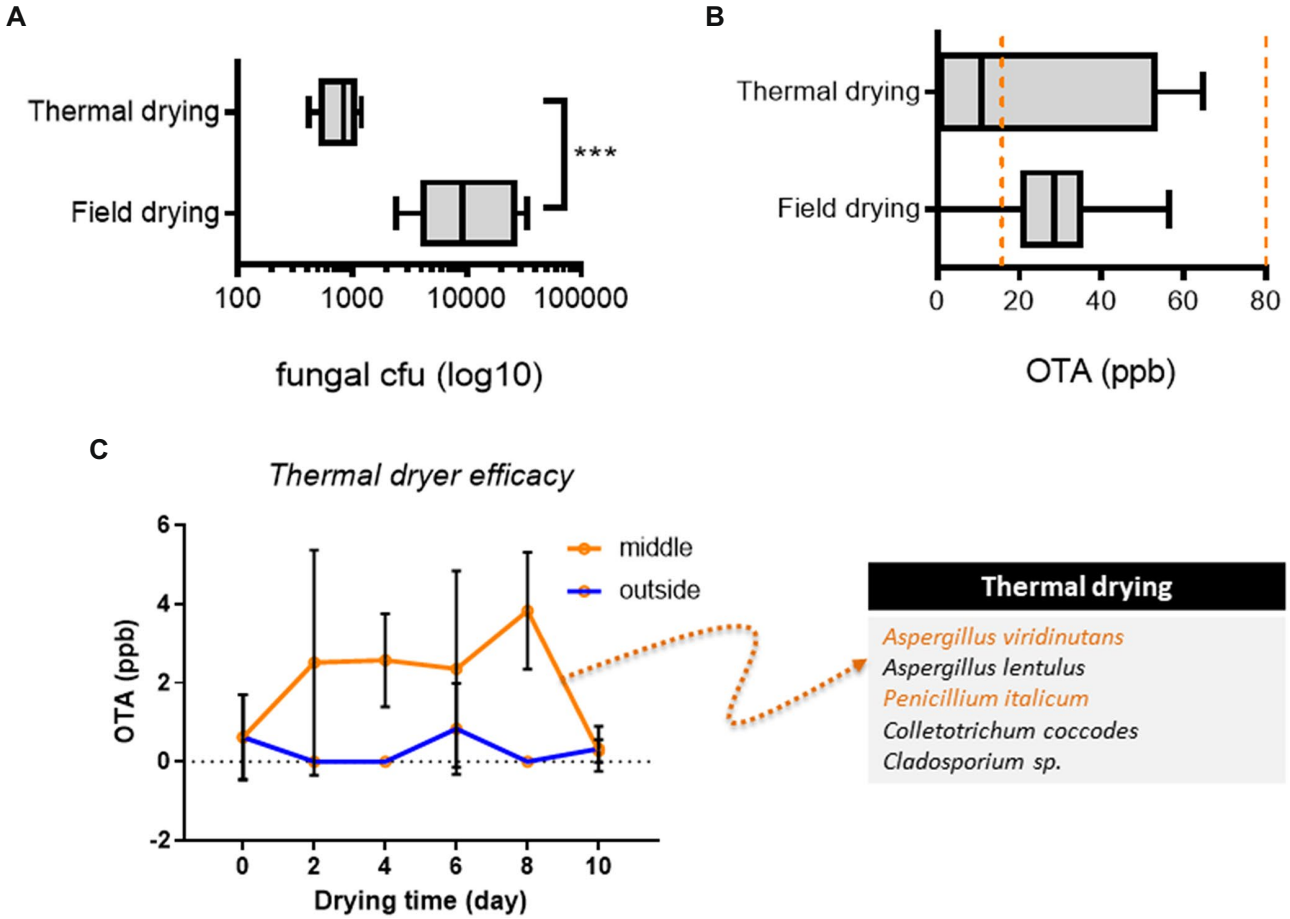
Analysis of thermal drying. **(A,B)** Fungal levels **(A)** and OTA **(B)** levels in AGR products from each drying (*n* = 5 per group). Results are shown as a box-and-plot with Tukey whiskers indicating the difference between the 75^th^ and 25^th^ percentiles with Student’s *t*-test. Asterisks indicate significant differences (***p < 0.001). The orange-colored dashed lines indicate the EU regulatory limits for OTA in medical crops (15–80 ppb). **(C)** OTA levels and fungal species (the left box) from different parts of AGR loads in the thermal dryer (*n* = 6 per group). Results are shown as the mean ± standard deviation (SD) with Student’s *t*-test (*p* = 0.05). AGR, Angelicae Gigantis Radix; OTA, ochratoxin A.

We investigated the drying process in terms of the fungal risk. In particular, field-drying (pCCP_1_) has the potential risk of greater fungal inoculation than thermal drying. As expected, thermal drying effectively reduced total levels of fungal inoculation (Figure 4A). However, some portions of products from the thermal drying process and most products from the field-drying process were contaminated with relatively high levels of OTA (15–80 ppm) although OTA levels in conventional practice-based products were less than the upper limit of EU guidelines (80 ppb, Figure 4B). The moisture contents in AGR products after the thermal drying were 10.09–13.72% while those after the field drying were 11.11–17.47% (Supplementary Figure 1B). However, all product moisture levels were maintained at less than 13.00% after the slice drying, which corresponds to the water activity of less than 0.7 in *Angelica* roots (Zhang et al., 2018). The thermal dryer was further investigated for OTA production, as this stage was assumed to be a provisional CCP (pCCP_3_). Conventionally practiced thermal drying was performed with a maximal AGR load. There was a marginal void space in the incubator to reduce the energy cost for small-scale farms (Figure 1B; pCCP_3_). The products were assumed to be dried under poor heat transfer conditions, which may allow fungal growth and toxin production. Therefore, OTA levels were compared between the inside of the AGR load and the outside load, which was close to heat outlets. Although the outside AGR load maintained marginally safe OTA levels during the drying process, the internal load showed a notable increase in OTA during the same process (Figure 4C). This result indicated that improperly performed thermal drying could allow mold growth and toxin production, although the thermal process of prolonged drying effectively retarded toxin production. Furthermore, fungal communities were analyzed in products obtained using the drying and storage conditions. Although we could detect *Phoma* and *Fusarium genera*, we focused on non-*Fusarium* mycoflora communities in terms of OTA production, which was typical in each treatment (Supplementary Figure 1C). Of note, after 2–8 days of thermal drying, products from the outside load were contaminated with several potent OTA producers, including *Aspergillus viridinutans* and *Penicillium italicum* (Figure 4C). Collectively, improper thermal drying (overload or short period of drying) was positively associated with OTA production and the growth of OTA-producing fungi, although the process effectively reduced the total mold levels in AGR products.

## Discussion

Improperly practiced postharvest procedures are associated with increased exposure to OTA during AGR production. In particular, three provisional CCPs, field-drying, soil removal, and thermal drying, were addressed in the conventional procedure. Compared with the conventional practice, the official protocol with an efficacy-proven washing process did not afford considerable advantages for reducing OTA production, indicating that post-washing steps, such as thermal drying, pose an additional risk for microbial contamination. Overloaded or insufficient thermal drying is an unknown provisional CCP that needs to be controlled to reduce the risk of toxin production. Due to these two hindrance factors, the thermal drying was not efficient in reducing the moisture contents as field drying in a short period. Optimal loading and sufficient drying time are required for safe AGR production. However, insufficient thermal transfer and overload allowed the growth of potent OTA producers in a mold-favorable moisture condition even during the thermal drying. In the case of the official method, the washing process posed a higher risk of mold growth than the non-washing protocol in addition to the bioactive component loss. Moreover, the drying temperature is another key factor to control the fungal growth and toxin production in AGR products. According to the official protocol, the recommended temperature of the thermal dryer is approximately 40°C which is predicted unfavorable for OTA production (Medina et al., 2017). Moreover, mold growth and toxin production are limited under high temperatures with low water activities (<0.8). However, the performance temperature of thermal dryers in many small-scale farms was less than 35°C in our unpublished survey, indicating inefficient control of OTA risk during AGR production. Therefore, CCP-based evaluations should be systematically regulated in terms of water activity and temperature during each process, which should be practically recognized by the workers and field educators.

To reduce energy costs, some farms perform field-drying for several months. However, field-drying is not recommended based on the present evaluation, given that AGRs are exposed to high microbial loads. Although freshly harvested products contain marginal OTA levels, prolonged outdoor exposure can increase the risk of fungal contamination, growth, and toxin production during the field-drying process. Moreover, morphological changes are known to frequently occur, including an oxidative blackish color change on the AGR surface after field-drying. Therefore, the CCP_1_-linked process needs to be avoided by carrying out thermal drying in a clean facility with a well-controlled temperature system. In contrast, field-dried products require a washing process to reduce total microbial loads, despite concerns regarding the loss of pharmacological components during washing. Based on the previous investigation, levels of nodakenin and total decursin are 3.56–6.13% in the AGR dry matter after the washing process compared to 4.36–8.34% in the products from the non-washing cleaning (Seong et al., 2018). Since the commercially accepted levels of nadakenin and total decursins in AGR are more than 4.00%, only 10% of washing-processed AGR products can satisfy the commercial guideline and most farms perform the conventional method without the washing process. It is thus warranted to develop an optimally mild washing or alternate cleaning technology to attenuate microbial levels in products.

Compared with conventional practice-prepared AGRs, the official protocol-based products with the washing process remain contaminated with high levels of OTA (>80 ppm [EU regulatory limit for herbal crop products]). This is possibly attributed to fungal contamination in later steps, such as thermal drying, which was poorly performed at the farm level. The amount of loaded product was occasionally very high to reach the optimal drying temperature, which allowed fungal growth, subsequently inducing a significant increase in mycotoxin production. In particular, the inner parts of the loaded product had a higher amount of OTA than the outer parts close to the machine heat outlet. Therefore, optimal loading and verifying the inner temperature should be adopted as a preventive step to reduce the risk of OTA exposure in AGRs. Moreover, additional low-cost drying technologies must be developed to satisfy the demands of small-scale farms.

Non-washed products can present diverse soil-borne microbial hazards, including fungal contamination and toxin production. We identified several potent OTA producers, including *A. viridinutans* and *P. italicum*. Some *Aspergillus* species, such as *A. ochraceus, A. alliaceus, A. sclerotiorum, A. sulphureus, A. albertensis, A. auricomus,* and *A. wentii*, are known to produce OTA (Varga et al., 1996). In particular, OTA-producing *A. viridinutans* strain is most closely related to an asexual isolate (Varga et al., 2000). Although *P. verrucosum* and *P. nordicum* are the only OTA-producing species currently accepted in the genus *Penicillium,* we identified a postharvest phytopathogen, *P. italicum,* which is also known to produce OTA (Vankudoth et al., 2016; Vaseghi et al., 2018). OTA-producing *P. italicum* can be controlled by pesticides such as imazalil, pyrimethanil, fludioxonil, and tiabendazole, but which are toxic to both the environment and humans (Kanashiro et al., 2020). In addition to toxigenic fungi, human pathogenic bacteria and viruses can be present in unwashed AGR products. For instance, enterohemorrhagic *Escherichia coli* and *Salmonella* species can survive in soil microenvironments for more than 12 weeks (Jechalke et al., 2019; Detert and Schmidt, 2021). In terms of mycoflora, the community was not static during the AGR production. Depending on the drying and storage conditions, the compositions of non-*Fusarium* species were typical (Supplementary Figure 1C). In the case of field drying, AGR products were not contaminated with OTA producers. One possibility of this regulation is that field environment-derived microbial communities might counteract the OTA producers in the following stages. Non-washing soil removal is not consistently harmful in terms of the microbial community. Some products that did not undergo washing displayed marginal levels of OTA or ochratoxigenic fungi. One potential reason for suppressing toxigenic fungi is microbial competition with the remaining soil microbes (Alabouvette et al., 2009). However, the washing process generally can provide a clean, noncompetitive environment for toxin producers. Since the washing process can facilitate the growth of toxin producers or other secondary invaders, safety issues associated with the washing process need to be carefully assessed in terms of the products’ next utility or fate in the environment. If non-washing soil removal is sufficient to control the subsequent microbes *via* a competitive microbial ecology, it could offer advantages over the washing process. Moreover, an ideal drying process with optimal product loads could attenuate the microbial risk from the non-washing process. As mentioned earlier, owing to the loss of pharmacological components, the regular washing process will not be preferred on production farms. Therefore, further management or technical support is needed to improve thermal drying and subsequent storage steps as future CCPs in AGR production.

## Data availability statement

Data are available upon request from the authors. The data supporting the findings of this study are available from the corresponding author upon reasonable request. Some data may not be available owing to privacy or ethical restrictions.

## Author contributions

Project design and hypotheses were defined by YM. YM, JK, and TA conducted experiments and analyzed the data. TA supported field sampling and fungal evaluations. YM prepared the manuscript and supervised the overall project. All authors contributed to the article and approved the submitted version.

## Funding

This research was supported by the Basic Science Research Program through the National Research Foundation of Korea (NRF) funded by the Ministry of Education (2018R1D1A3B05041889 and 2022R1I1A1A01065276).

## Conflict of interest

The authors declare that the research was conducted in the absence of any commercial or financial relationships that could be construed as a potential conflict of interest.

## Publisher’s note

All claims expressed in this article are solely those of the authors and do not necessarily represent those of their affiliated organizations, or those of the publisher, the editors and the reviewers. Any product that may be evaluated in this article, or claim that may be made by its manufacturer, is not guaranteed or endorsed by the publisher.
